# Predicting parasitic plants *Loranthus Europaeus* range shifts in response to climate change

**DOI:** 10.1038/s41598-025-03631-2

**Published:** 2025-05-29

**Authors:** Marlena Baranowska, Adrian Łukowski, Robert Korzeniewicz, Wojciech Kowalkowski, Łukasz Dylewski

**Affiliations:** 1 Faculty of Forestry and Wood Technology, Wojska Polskiego 71A, 60-637 Poznań, Poland; 2 Faculty of Veterinary Medicine and Animal Science, Poznań University of Life Sciences, Wojska Polskiego 71C, 60-637 Poznań, Poland

**Keywords:** Bioclimatic variables, Climate scenarios, MaxEnt, Mistletoe, Oak, Species distribution modeling, Climate sciences, Climate change, Climate and Earth system modelling, Ecological modelling, Ecology, Forest ecology

## Abstract

Climate change significantly influences the distribution of parasitic species, posing threats to ecosystems and economies. This study examines the potential range expansion of *Loranthus europaeus*, a parasitic plant impacting European forestry. We assessed the impact of predicted climate change for 2041–2060 and 2061–2080 using MaxEnt modeling based on current occurrence data of *L. europaeus*, and the main host plant genus oak *Quercus*, as well as bioclimatic variables. Our model demonstrated high predictive accuracy (AUC = 0.92). The most important variables for Europe range were range of *Quercus* genus. Key environmental factors included isothermality (bio3) and mean temperature of wettest quarter (bio8). Under SSP126 and SSP245 scenarios, our results predict significant range expansions into northern and eastern Europe, with increases of 43.5% and 53.9% by 2041–2060. Conversely, southern Europe may see contractions of 16.4–20.6%. Projections for 2061–2080 indicate further expansions up to 65.8% in northern Europe, alongside contractions up to 29.8% in southern regions, including Turkey and Greece.These shifts highlight the influence of climate change on *L. europaeus* distribution and underscore the need for adaptive management strategies to mitigate potential ecological and economic impacts.

**Supplementary Information:**

The online version contains supplementary material available at 10.1038/s41598-025-03631-2.

## Introduction

Climate change significantly influences the geographic ranges of plant species, with profound implications for biodiversity conservation^1–3^. Species migration in response to climate change depends on factors such as life history traits and dispersal syndromes^4,5^. Evidence from pollen and subfossil materials indicates that even after thousands of years, plant species have not fully occupied their potential ranges due to dispersal limitations^2,6–8^. However, current climate change rates pose unprecedented risks of biodiversity loss. Climate change acts as a potent force driving the proliferation of alien and pest species, intensifying ecological disruptions, and presenting formidable challenges to ecosystems globally^9,10^. Fluctuations in temperature, precipitation patterns, and extreme weather events create conducive environments for non-native species to establish and spread into new territories^10,11^. Moreover, climate alterations weaken historical barriers that once constrained the expansion of these species, accelerating their range shifts^12^.

Recent advancements in climate modeling have forecasted rapid temperature increases alongside a heightened frequency of extreme weather events such as droughts, cold snaps, and heatwaves, coupled with localized reductions in rainfall^13^. These ongoing shifts in climate dynamics are driving alterations in the geographical ranges of organisms, necessitating adaptations to new environmental conditions. Consequently, these changes are reshaping spatial distributions and compelling species to adjust to novel climatic niches. Projections suggest that many species will undergo distributional shifts towards higher elevations and latitudes in response to escalating temperatures^12^. Furthermore, climate change is altering the dynamics between plants and their biotic stressors, leading to an expansion in the range of pathogens and pests. This phenomenon increases the vulnerability of forests to ensuing stresses, heightening their susceptibility to disturbances related to climate change^14^. Trees and forests, known for their long-term functionality, are particularly at risk. Understanding these evolving dynamics is crucial for effective species and habitat management^12^, as well as for planning future forest management strategies^15,16^. Many biotic and abiotic factors were not considered significant for forest management^17^. One such factor is the common mistletoe (*Viscum album* L.), a semi-parasitic plant whose economic impact as a forest pathogen, particularly among coniferous trees, has been overlooked. Previously, mistletoe was regarded as enhancing forest biodiversity^18^. However, there is now a notable increase in the presence of *V. album* in European forests, leading to increasingly severe losses^19^. Modeling conducted by Walas et al.l^19^ suggests that the potential range of mistletoe is shifting north-eastward, with mountainous regions experiencing elevational shifts. This expansion of mistletoe presence poses a significant threat to pine-dominated forests, especially in Central and Eastern Europe, potentially accelerating tree dieback.

Another mistletoe species found in Europe is *Loranthus europaeus* (Jacq.) (Santalales: Loranthaceae)^20^. This mistletoe parasitizes various forest tree species but poses a particular risk to oaks^21^. Currently, *L. europaeus* is widely distributed across southwestern Europe, southern Russia, Anatolia, Iran, Iraq, and isolated areas in Asia Minor and Ukraine^22–24^. Its presence has also been confirmed in Germany, particularly in relict sites in Saxony where favourable warm air currents from the Elbe valley aid its establishment^25^. It has been observed mainly on various species from oak *Quercus* spp. L. genus and less frequently on sweet chestnut *Castanea sativa* Mill., but also has been occasionally found on other woody plants like *Acer campestre* L., *Betula pendula* Roth., *Carpinus betulus* L., *Colutea arborescens* L., *Crataegus monogyna* Jacq., *Fagus sylvatica* L., *Fraxinus ornus* L., *Prunus spinosa* L., *Robinia pseudoacacia* L. and *Olea europaea* L^21,26–34^. Research by Kubov et al.l^35^ highlights that the invasion of *L. europaeus* poses a serious threat to the physiology and growth of sessile oaks (*Quercus petraea* (Matt.) Liebl.). Infected trees show a 20–30% reduction in growth, increased crown dieback, and heightened susceptibility to pathogens, particularly in older stands, thereby compromising forest durability and productivity amidst changing climates. Forest protection procedures against *L. europaeus* are currently undefined due to the plant’s specificity, population dynamics influenced by climate change, and its interactions with birds. Numerous bird species, such as the mistle thrush (*Turdus viscivorus* L.)^36^, the Bohemian waxwing (*Bombycilla garullus* L.)^37^, and the Eurasian jay (*Garrulus glandarius* L.)^38^, feed on its fruit and aid in its dispersal over long distances^34,39^. For instance, Slovakia lacks specific protection measures and emphasizes the need for ongoing monitoring^35^ Understanding the phenology of *L. europaeus* is crucial for its control^40^, especially given potential shifts in its flowering and fruiting patterns due to climate change, impacting animal migrations and activities^41^. Climate changes could potentially expand the distribution of mistletoe^32,42,43^, possibly doubling its population within 16 years due to early maturation—fruiting as early as 3 years old^44^. Frost days per year, with an isotherm of 110 days, may limit mistletoe expansion in the Czech Republic and Moravia^45^, with late spring frosts also posing constraints^46^. *Loranthus europaeus* currently thrives in southeastern Europe due to its high heat requirements, but its northward migration is anticipated along valleys such as the Danube, Inn, and Elbe with rising temperatures^47^.

Despite the comprehensive documentation available regarding the presence and abundance of *L. europaeus*, primarily derived from short-term studies^48–52^, as well as predictions of its local spread^32,34,43,49,53,54^, there remains a notable absence of research examining the potential future Eurasian range of this species. Such studies are imperative to understand the dynamics of its spread, especially concerning forests vulnerable to this semi-parasitic plant, particularly in the context of climate change. Therefore, we hypothesized that this species could potentially benefit from projected climate changes and might pose a future threat to forest health in Europe. Hence, the objective of this analysis was twofold: firstly, to assess the current and projected future occurrence range of *L. europaeus* in Europe during the 2040–2060 and 2060–2080 periods, and secondly, to underscore the urgent need for predictive modeling to inform effective forest management strategies aimed at mitigating the adverse impacts of this species, exacerbated by changing climatic conditions.

## Materials and methods

### Occurrence data

**Fig. 1:**
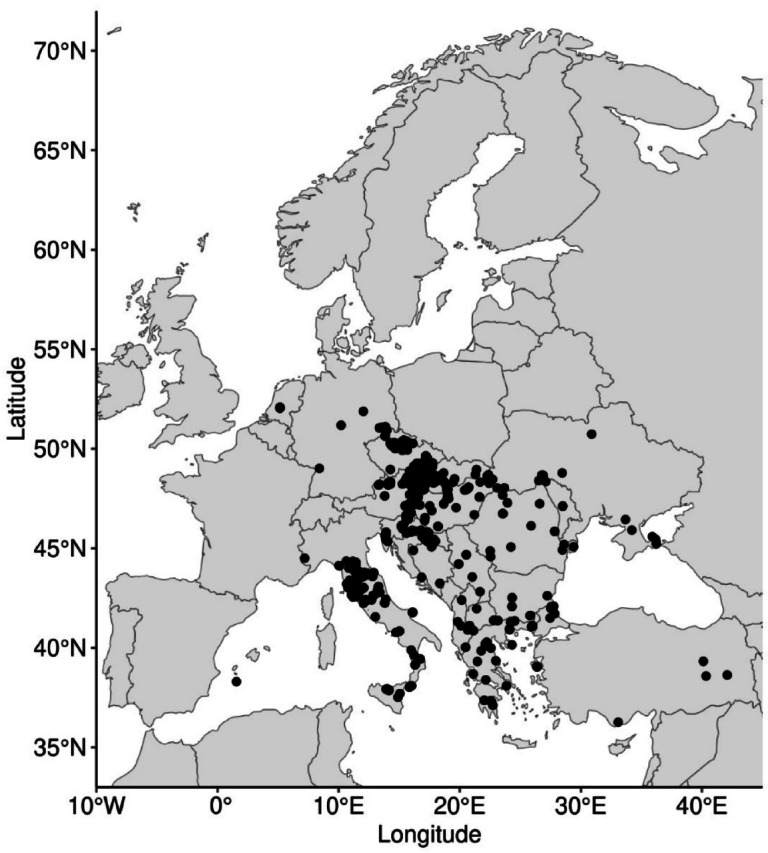
Observed distribution of *Loranthus europaeus* in Europe retrieved from Global Biodiversity Observa-tion Facility (GBIF.org, 2024), field surveys, herbarium and literature records.

To model the potential distribution of *L. europaeus*, we collected occurrence data from various sources (A.1.), including field surveys, herbarium and literature records, and online databases such as GBIF (Global Biodiversity Information Facility). The dataset was curated to remove duplicates and ensure accuracy, resulting in a comprehensive dataset of georeferenced occurrence points across Europe (Fig. 1). This dataset provided the foundation for modeling the species’ current and future distribution patterns. In total, we collected 997 distribution records for *L. europaeus* and 49,684 for *Quercus* genus, each with geographical coordinates (latitude and longitude), and entered them into a spatially referenced database.

To reduce sampling bias caused by uneven data density across regions where some areas are over-sampled and others under-sampled^56^, we retained only one randomly selected occurrence per species within each 0.25° grid cell. This approach, commonly used in previous studies^57–59^, helped ensure a more balanced spatial representation of species and regions in the dataset compared to the original data input.Following resampling, the dataset comprised 2631 occurrences for *Quercus* spp. and 290 for *L. europaeus*, which were deemed sufficient for MaxEnt analyses^60^. The analyses covered the area of Europe, limited by longitude from − 10° to 45° and latitude from 33° to 72°. Utilizing presence-only data, we employed the MaxEnt algorithm to model species distributions. This method relies on pseudoabsences instead of absence data, with predictions based on comparing presence patterns to background data^61,62^. By including background points, the model becomes more conservative, requiring stronger signals to counteract the presence of background points^61^.

### Predictors of the potential distribution

Initially, we employed all 19 bioclimatic variables to model the global distribution of *Quercus* species as described by Dyderski et al.l^58^. In the context of large-scale species distribution modeling, it is deemed appropriate to incorporate all bioclimatic variables^63–65^. Subsequently, for *L. europaeus*, we utilized a reduced set of bioclimatic variables following an assessment of collinearity among them. Multicollinearity was mitigated by eliminating variables exceeding collinearity thresholds of |r| > 0.7. A total of five bioclimatic variables, along with the predicted distribution of *L. europaeus*, were considered for model development (Table 1). The raster resolution utilized in the analyses was set at 2.5’. These bioclimatic variables were sourced from the WorldClim 2.1 database (www.worldclim.org), providing high-resolution climate data necessary for accurate species distribution modeling^66^.

**Table 1:** Bioclimatic variables used in the potential distribution modeling of *Loranthus europaeus*. The finally used in the analysis maps were marked with bold.

Bioclimatic variables	Abbreviation
Annual mean temperature	bio1
Mean monthly temperature range	bio2
Isothermality ((bio2/bio7) × 100)	**bio3**
Temperature seasonality (standard deviation × 100)	bio4
Max temperature of warmest month	bio5
Min temperature of coldest month	bio6
Temperature annual range (bio5–bio6)	bio7
Mean temperature of wettest quarter	**bio8**
Mean temperature of driest quarter	bio9
Mean temperature of warmest quarter	bio10
Mean temperature of coldest quarter	bio11
Annual precipitation	bio12
Precipitation of wettest month	bio13
Precipitation of driest month	bio14
Precipitation seasonality (coefficient of variation)	**bio15**
Precipitation of wettest quarter	bio16
Precipitation of driest quarter	bio17
Precipitation of warmest quarter	**bio18**
Precipitation of coldest quarter	**bio19**

### Potential niche modeling

We used MaxEnt (Maximum Entropy Modeling) to predict the current and future potential distribution of *L. europaeus*. MaxEnt is a widely used method for species distribution modeling due to its robustness in handling presence-only data and its ability to provide accurate predictions. The analysis was conducted using default settings, with 80% of resampled data points allocated to the training set and 20% to the validation set. For each species, we selected 10,000 pseudoabsences (background points). The quality of the MaxEnt model was assessed using the AUC (area under the receiver operator curve), which measures the overlap between true and predicted occurrences. The MaxEnt analysis generated raster maps indicating the probability of each species occurring in each grid cell. Threshold values for presence/absence maps were calculated to optimize sensitivity (proportion of true positives) and specificity (proportion of true negatives), aiming to balance false negatives and false positives^67^. Additionally, MaxEnt provided the percentage contribution of variables to the potential distribution model.

Data analysis was performed using R software (R Core Team,^68^, with MaxEnt models developed using the ‘dismo’ package^69^ For geospatial analyses and data processing, we utilized the ‘raster’^70^ and ‘sf’^71^ packages. The potential range saturation, indicating the proportion of sampled points and grid cells suitable for species occurrence, was calculated as a result.

### Predicted changes in species distributions

Our future climate projections are derived from the IPCC’s 6th Assessment Report and the Shared Socioeconomic Pathways (SSPs) elucidated by Riahi et al. (2017). The utilization of SSPs serves to account for uncertainties in potential mitigation strategies. Specifically, we employed four SSPs: SSP126 (representing sustainability, akin to RCP2.6), SSP245 (illustrating a moderate, middle-of-the-road scenario akin to RCP4.5), SSP370 (characterizing regional rivalry, absent in the 5th report), and SSP485 (depicting fossil fuel-based development or business-as-usual, akin to RCP8.5). Across these SSPs, data from four global circulation models (GCMs) were incorporated: IPSL-CM6A-LR (France), MRI-ESM2-0 (Japan), CanESM5 (Canada), and BCC-CSM2-MR (China). Our predictions were based on maps corresponding to future climate scenarios. To mitigate uncertainty associated with particular General Circulation Models (GCMs), we averaged the predicted climatic suitability for each Shared Socioeconomic Pathway (SSP) across the four GCMs under study^72,73^. By applying threshold values (true/false) on the maps, we calculated potential distribution shifts by adjusting values from 1 to 2. Subsequently, we performed the following calculation to estimate potential range changes: (i) areas unsuitable for colonization in all climate scenarios (0–2*0 = 0); (ii) areas optimal for colonization under future climatic conditions (0–2*1=-2); (iii) currently optimal areas projected to be lost in the future (1–2*0 = 1); (iv) currently optimal areas expected to remain suitable in the future (1–2*1=-1)^59^.

## Results

The *Quercus* genus potential distribution had moderate performance expressed by AUC 0.81 predicted for Europe assessed using a validation dataset. The threshold of occurrence probability, assessed as the point with the highest sum of sensitivity and specificity, was at 0.57. The most important variables for *Quercus* genus in Europe were bio7 (45.6%), bio1 (20.9%) and bio4 (15.8%) (B. 1.).

**Fig. 2:**
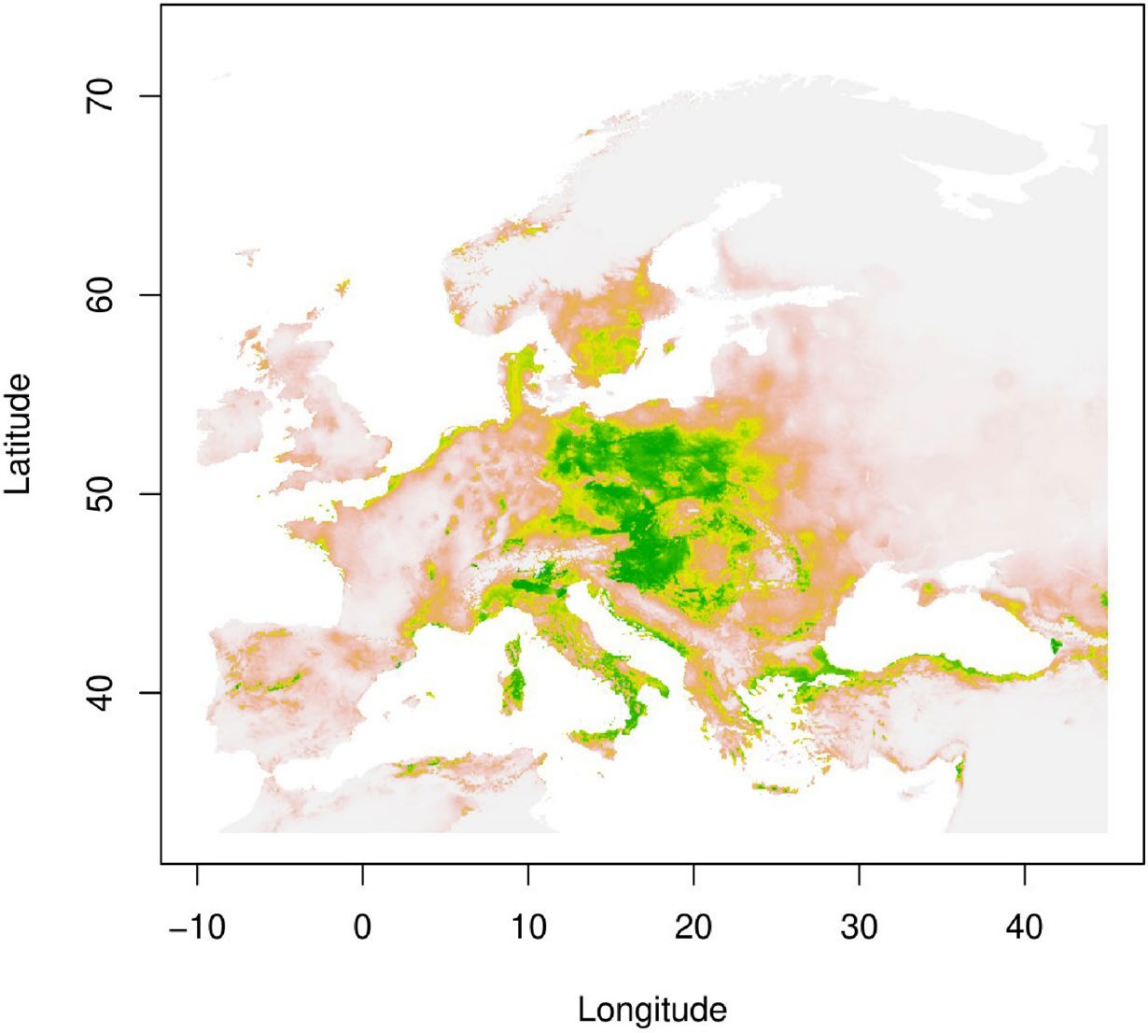
Predicted climatic suitability for *L. europaeus* using the climatic data. According to the maximum specificity and sensitivity, pixels with climatic suitability > 0.36 are claimed to be within the predicted range.

The *L. europaeus* potential distribution had good performance expressed by AUC 0.92 predicted for Europe assessed using a validation dataset. The threshold of occurrence probability, assessed as the point with the highest sum of sensitivity and specificity, was at 0.37. The most important variables for Europe range of *L. europaeus* were bio3 (41.2%), probability of occurrence *Quercus* ssp. (30.5%) and bio8 (20.5%). The rest bioclimatic variables explain in total 7.8% (bio15–4.7%, bio18–1.6%, bio19–1.5%). Our analysis uncovered that although the majority of current *L. europaeus* habitats, notably in the Czech Republic, Hungary, Austria, and Italy, are situated within favourable climatic conditions (Fig. 2). Notably, the Poland, east Germany, Slovakia, Denmark, the southern part of Sweden and some eastern part of Belarus, and Ukraine also represent suitable environments for *L. europaeus* expansion. Conversely, areas in far northern-east Europe, Portugal, southern Spain, southern Turkey, and Greece fall outside the range of climatic suitability.

**Fig. 3:**
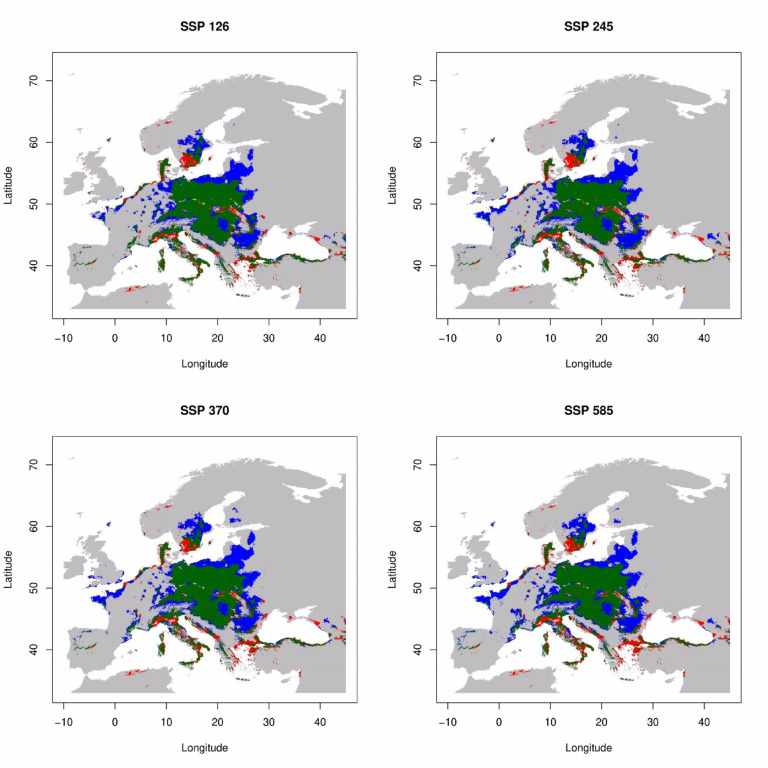
Current and projected ranges of *L. europaeus* in 2041–2060 timeline. Explanations: green + red – current potential distribution, green – persistence, red – future range contraction, blue – future range expansion, grey – still unsuitable.

**Fig. 4:**
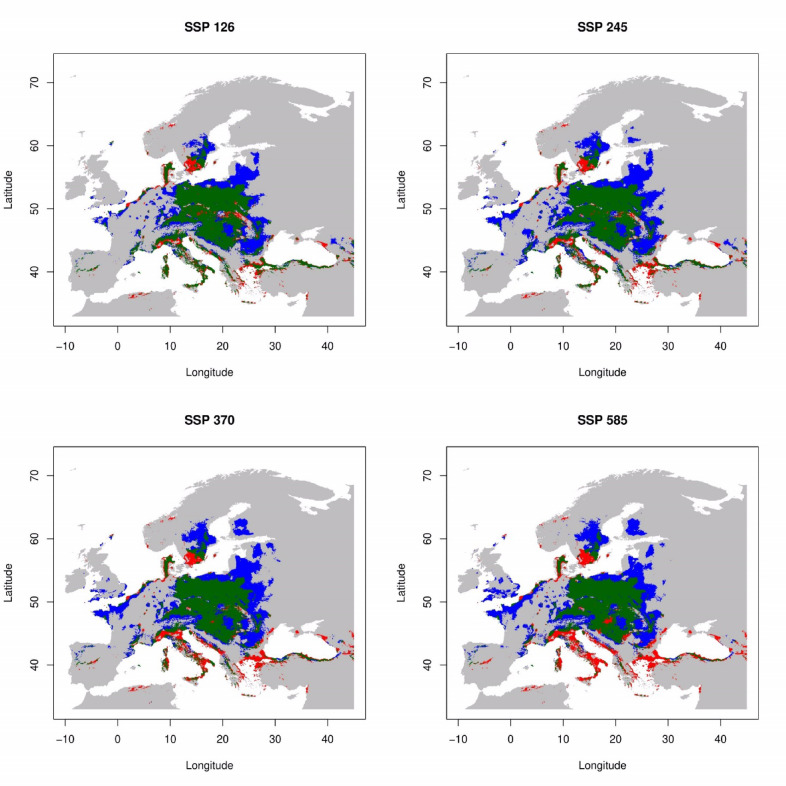
Current and projected ranges of *L. europaeus* in 2061–2080 timeline. Explanations: green + red – current potential distribution, green – persistence, red – future range contraction, blue – future range expansion, gray – still unsuitable.

**Fig. 5:**
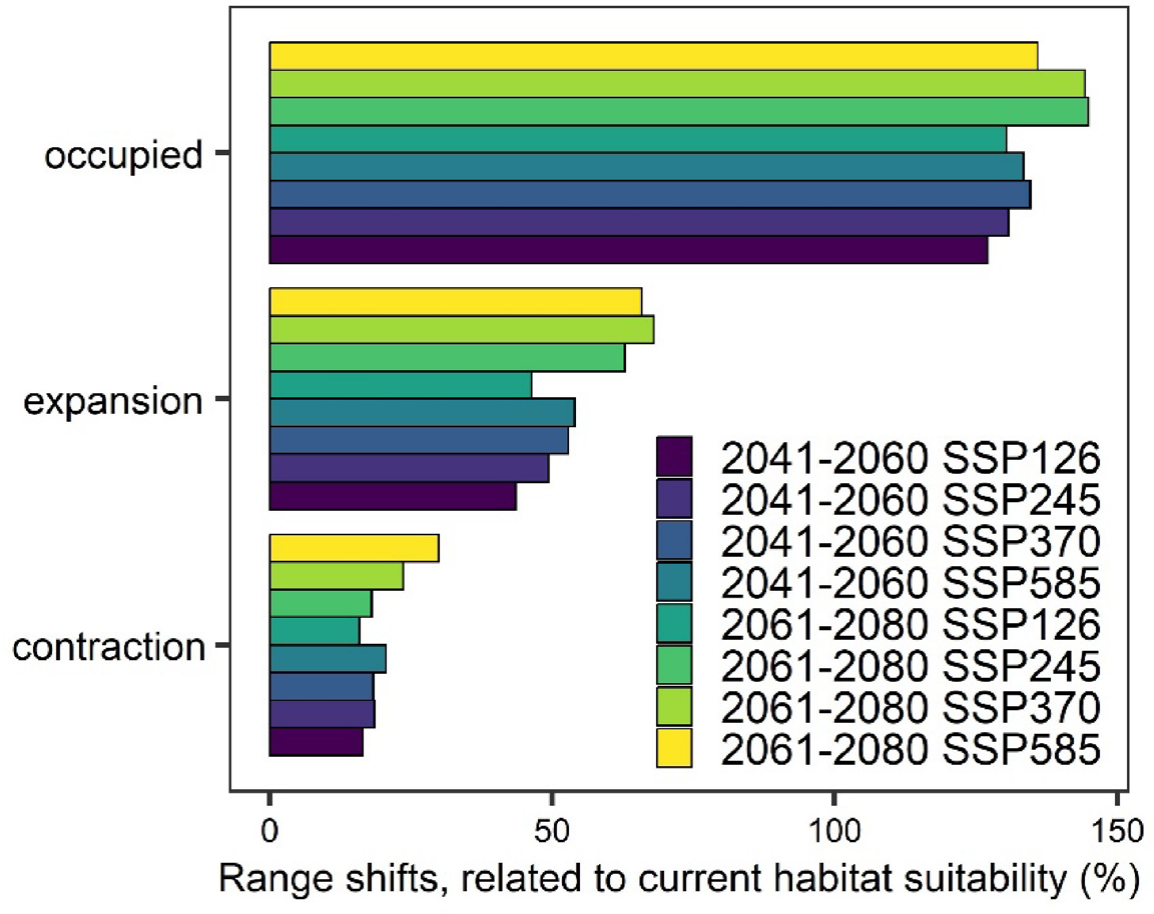
Range shifts of *L. europaeus* in four scenarios and two timelines of the climate change.

Under the whole scenario, the species are expected to remain present in most regions during period 2041–2060 (Fig. 3) and 2061–2080 (Fig. 4). Predictions for the 2041–2060 scenarios of SSP126 and SSP245 indicate a range expansion of 43.5% and 53.9% (Fig. 5), respectively, in northern Europe (northern Poland, and Baltic state), and central and east part of Europe, especially Ukraine and Romania (Fig. 3). Conversely, there is a projected range contraction of 16.4% for SSP126 and 20.6% for SSP585 in the southern part of Europe, particularly in the Balkans and Italy, as well as in the southern regions of Sweden (Fig. 3). Projection for 2061–2080 timeline indicated significant range expansion from 46.3% for SSP126 to 65.8% for SSP585 is anticipated (Fig. 5), particularly in northern Europe, including Finland and Russia, and in the northeast part of Europe, notably in northern France and the UK (Fig. 4). Concurrently, range contraction under SSP126 to SSP585 scenarios is estimated to be between 15.9 and 29.8% (Fig. 5), concentrating in the southern parts of Europe, such as Italy, the Balkans, and extending into Turkey (Fig. 4).

## Discussion

Our study presents potential species distribution range for the ecologically and economically significant parasite *Loranthus europaeus*. Previous research^30,32,40,54,74,75^ has highlighted its impact, but our results represent the first comprehensive modeling of its distribution across the entire European range. *Loranthus europaeus*, known for its westward migration to Europe, currently thrives in Central Asia, Anatolia, South Russia, and Southwestern Europe^22^. Our findings indicate that *L. europaeus* could potentially expand further, which has significant implications for local ecosystems and economies. Its impact on the forestry economy is particularly notable, as it can cause dieback in host tree species, leading to substantial economic losses. The dieback of host trees not only reduces timber yield but also affects biodiversity and forest health, underscoring the need for effective management strategies to mitigate the spread and impact of this parasitic species. The extent and impact of damage caused by *L. europaeus* have been evaluated locally in various regions. For example, in Iran, up to 78% of forest trees are affected by this mistletoe^54^. In Slovakia, during the 1980s, mistletoe infestation covered 34% of oak forest areas^74^. In Kosovo, the presence of *L. europaeus* in sessile oak stands reached 14.65%^33^. In Croatia, around 7% of oak populations were infested in 2002/2003^31^, while in Turkey, only 2.3% of sessile oaks were affected^31^, in Podyjí National Park, Czech Republic (2011–2015) 6.9–9.7% of *Q. petraea* shoots were colonized by *L. europaeus*^53^. Sayad et al.^52^ reported that the infection rate in the oak forests in Zagros in Irak was 23%. Additionally, Ilić^76^ indicates that *L. europaeus* has become a new problem in the management of oak stands, a situation exacerbated by climate change in the Motajica Mountains of Bosnia and Herzegovina. The results of our research, alongside the aforementioned studies, demonstrate that the occurrence of *L. europaeus* is not only a local issue. We anticipate an expansion in the range of this species, which underscores the growing concern.

The distribution of *L. europaeus* is influenced by more than just the presence of its host; other factors can also limit its occurrence. Our results indicate that environmental conditions are likely the most significant factors shaping the range of *L. europaeus*. The climatic variables with the highest impact on potential distribution models for *L. europaeus* are the mean temperature of the wettest quarter (bio8), isothermality (bio3), and host plant distribution. MaxEnt modeling, previously used by Walas et al.l^19^ to predict the range of another parasitic plant, *Viscum album*, suggests that temperature is the key variable, while precipitation is less important. This is likely because rainfall has an indirect impact, given that *L. europaeus*, like *V. album*, extracts water from the xylem of its host. This underscores temperature as the most critical atmospheric factor influencing the distribution of *L. europaeus*. The anticipated increase in average annual temperature is predicted to facilitate the further spread of this species in the coming decades^47,77^. The northern limit of the geographical distribution of mistletoe has been primarily influenced by climatic conditions, particularly winter and spring frosts^46^. According to Rejmánek^45^, the average number of frosty days per year is a limiting factor for the occurrence of *L. europaeus*, with the isotherm of 110 frosty days per year restricting its spread.

Currently, the actual range of *L. europaeus* is considerably smaller than that of its primary host, *Quercus* sp. in Europe. Numerous examples demonstrate that the presence of *L. europaeus* in forests poses a significant threat to forest durability and productivity in the context of climate change. This species, akin to *Viscum album* subsp. *austriacum*, likely survived the glacial period in the Iberian Peninsula and subsequently recolonized other parts of Europe from this region^19^. However, there remains potential for the expansion of *L. europaeus*, as its current estimated range exceeds its observed distribution. Our model for the occurrence of *L. europaeus* resembles the predicted range of *V. album* subsp. *austriacum* as described by Walas et al.l^19^. The reduced range of *L. europaeus* in the north may be attributed to its heightened sensitivity to lower temperatures and specific terrain features, such as those along the borders of Poland and Slovakia, and Poland and the Czech Republic. The harsh climates of the Sudetes and Carpathian Mountains could also act as barriers to the spread of *L. europaeus*. The anticipated distribution patterns are derived from correlation models based on environmental variables^61,78^, which may not always directly correspond to the physiology and ecology of the species under study^66^. Bioclimatic variables and their projections do not encompass extreme weather events, such as late spring frosts or summer droughts, whose occurrence, albeit unpredictable, is on the rise due to climate change. These events have the potential to impede the reproductive success of plants by causing damage to their generative organs, resulting in decreased fitness and heightened mortality rates, a phenomenon more extensively documented in trees^79–82^.

Future projections indicate that populations of *Loranthus europaeus* in the southernmost regions of the Balkan Peninsula may face extinction; however, the decline in suitability is projected to be less rapid in southwestern Europe. This variation can be attributed to the significant influence of continentalism on the distribution of this subspecies. Concurrently, the potential range is expected to remain relatively stable in the central part of Europe, with some countries such as Belarus, Germany, and Poland likely experiencing even higher suitability than at present, possibly due to anticipated increases in winter temperatures. Our findings suggest that akin to *V. album* subsp. *austriacum*, *L. europaeus* could become a substantial factor negatively impacting forests in Central Europe^19^. Sayad et al.l^52^ have a similar opinion, claiming that as a result of ongoing climate change and fragmentation of tree stands, L. europaeus has the potential to be more numerous and widespread in the forests of western Iran (Zagros).

Under current conditions, *L. europaeus* already presents a significant challenge in oak stands and chestnut plantations^51^, and this problem may be exacerbated by climate change, which could weaken host trees^19^. The presence of *L. europaeus* notably exacerbates tree stress during dry summer seasons, as infestation by this species reduces water and mineral nutrient availability. Mistletoe exhibits higher stomatal conductance and transpiration rates than the host tree, leading to a notable loss of water and affecting tree growth^83–85^. Affected hosts exhibit a 20–30% reduction in growth due to *L. europaeus* infestation^75^. This issue primarily affects older stands^32^. Additionally, Dolezal and Klimešová^79^ observed over a 50% increase in crown dieback in pedunculate oak *Quercus robur* L. trees with more than five *L. europaeus* individuals per tree.

*Loranthus europaeus* may be a primary factor predisposing oak trees, in particular, to the adverse effects of drought, initiating a decline process in which additional stressors, such as pests and diseases, could eventually lead to tree mortality. Mistletoe weakens oaks, resulting in gradual crown decline, especially in stands over 50 years old, rendering them more vulnerable to pathogens such as *Armillaria* spp. (Fr.) Staude or *Ophiostoma* spp. Syd. & P. Syd^86^. The potential range of the *L. europaeus* may also be influenced by human activity. Potentially, an increase in the abundance of *L. europaeus* is associated with the planting of its hosts. For example, in Poland, the reconstruction of coniferous (pine) forests in favour of deciduous species, such as oaks^87–89^, may facilitate the spread of this mistletoe. Additionally, the Common Agricultural Policy promotes the introduction of buffer strip and the creation of agroforestry systems^90,91^ with a dominant share of deciduous trees, including those species susceptible to *L. europaeus*. All these activities will increase the availability of host plants, which is a key factor in the distribution of *L. europaeus*.

Furthermore, the condition of oak stands in Poland has been deteriorating year by year. Oak decline, a complex process leading to increased mortality of this species, has been observed in Europe for many years. Previous studies suggest that climate conditions, especially drought, may be one of the most important factors triggering this phenomenon^92^. On the other hand, it is forecasted that the range of many oak species will change, with the range of many species expanding northward^93^, which is also confirmed by the results of our modeling of the range of trees belonging to the *Quercus* genus. Climate change will also favour the development of populations of the alien oak species in Europe, *Quercus rubra* L^94^, which is also susceptible to *L. europaeus*. The weakening of native oak populations, changes in their range, and the increase in the area of alien species may promote the expansion of the range of *L. europaeus* northward.

## Recommendations

The findings presented in this study provide insight into the expected changes in the distribution of *L. europaeus*. The potential range of *L. europaeus* is projected to shift northward and to higher altitudes in mountainous regions of Europe. This shift may have detrimental effects on forest ecosystems in Central and Eastern Europe, potentially accelerating the decline of deciduous tree species, mainly from genus *Quercus*. Conversely, southern populations of mistletoe may face local extinction. These projected range changes are primarily informed by climatic data; however, it is important to note that other factors not included in modeling such as seed dispersal by birds and pollination by insects also influence mistletoe spread. Nonetheless, our understanding of *L. europaeus* seed dispersal and germination remains limited, highlighting the need for further research in this area.

## Electronic supplementary material

Below is the link to the electronic supplementary material.

Supplementary Material 1

Supplementary Material 2

## Data Availability

The research was based partially on the following datasets from the GBIF database: GBIF.org (3 March 2024) GBIF Occurrence Download 10.15468/dl.f3m9gq.
