# Supplementary material for: Predicting parasitic plants Loranthus Europaeus range shifts in response to climate change
Source: Sci Rep. 2025 May 29;15:18932. doi: 10.1038/s41598-025-03631-2 (PMC12122712; doi:10.1038/s41598-025-03631-2)
Supplement: Supplementary file 1 — Supplementary Material 1 [file 41598_2025_3631_MOESM1_ESM.docx]

Table B.1. Percent contribution of bioclimatic variables in species distribution of *Quercus* genus in Europe.

| **Bioclimatic** | **Code** | **Percent contribution [%]** |
| --- | --- | --- |
| Annual mean temperature | bio1 | 20.9 |
| Mean monthly temperature range | bio2 | 1.2 |
| Isothermality ((bio2/bio7) × 100) | bio3 | 1.6 |
| Temperature seasonality (standard deviation × 100) | bio4 | 15.8 |
| Max temperature of warmest month | bio5 | 0 |
| Min temperature of coldest month | bio6 | 2.7 |
| Temperature annual range (bio5–bio6) | bio7 | 45.6 |
| Mean temperature of wettest quarter | bio8 | 1.9 |
| Mean temperature of driest quarter | bio9 | 0.6 |
| Mean temperature of warmest quarter | bio10 | 1.4 |
| Mean temperature of coldest quarter | bio11 | 0.1 |
| Annual precipitation | bio12 | 5.2 |
| Precipitation of wettest month | bio13 | 0.1 |
| Precipitation of driest month | bio14 | 0 |
| Precipitation seasonality (coefficient of variation) | bio15 | 0.2 |
| Precipitation of wettest quarter | bio16 | 0 |
| Precipitation of driest quarter | bio17 | 2.6 |
| Precipitation of warmest quarter | bio18 | 0 |
| Precipitation of coldest quarter | bio19 | 0.1 |
